# Risk of new-onset inflammatory bowel disease in psoriasis patients treated with five different interleukin inhibitors: a systematic review and meta-analysis

**DOI:** 10.3389/fimmu.2025.1594998

**Published:** 2025-06-04

**Authors:** Jia-Xin Zhang, Wen-Wei Li, Long-Zhuan Huang, Sha Lai, Zhi-Kun Qiu

**Affiliations:** Key Specialty of Clinical Pharmacy, The First Affiliated Hospital of Guangdong Pharmaceutical University, Guangzhou, China

**Keywords:** interleukin inhibitors, psoriasis, inflammatory bowel disease, meta-analysis, ixekizumab, risk difference, diarrhea

## Abstract

**Background:**

Interleukin inhibitors represent a standard therapeutic approach for psoriasis. However, there is still debate about the risk of new-onset inflammatory bowel disease (IBD) in psoriasis patients following interleukin inhibitor treatment. This systematic review and meta-analysis aims to evaluate the risk of new-onset IBD in psoriasis patients treated with five interleukin inhibitors (Bimekizumab, Ixekizumab, Secukinumab, Brodalumab, and Ustekinumab), providing insights to inform clinical decision-making.

**Method:**

This study was registered in the PROSPERO with registration number of CRD42024608423. The databases PubMed, Embase, Cochrane Library, and Web of Science were comprehensively searched for observational studies published as full-length papers in English. The Mantel-Haenszel method with a fixed-effects model and risk difference was used to compare the risk of new-onset IBD between experimental groups (using interleukin inhibitors) and the control groups (using placebo or non-interleukin inhibitors). Sensitivity analysis was performed using the leave-one-out method for the meta-analysis. Additionally, considering the potential for underdiagnosis of IBD, a meta-analysis of the risk of diarrhea was conducted.

**Result:**

This study included 17 articles covering 21 Randomized Controlled Trials(RCTs). A total of 22 new-onset IBD cases were reported in the experimental groups, with 3, 14, 4, 1, and 0 cases in the Bimekizumab, Ixekizumab, Secukinumab, Brodalumab, and Ustekinumab group, respectively. The control group only reported 1 case of new-onset IBD. No significant difference in the risk of new-onset IBD was found between these experimental groups and control groups. Based on the fixed-effects model, the pooled risk difference for Ixekizumab group was MH RD 0.0027 (95% CI 0.0001-0.0054, I² = 0%, P = 0.04). Sensitivity analysis indicated that the data was stable. Regarding diarrhea, a total of 95 cases were reported in the experimental groups, compared to 50 cases in the control groups. The experimental groups of Bimekizumab, Secukinumab, and Brodalumab reported 49, 45, and 1 case of diarrhea, respectively, while their control groups reported 11, 39, and 0 cases, respectively. Based on the fixed-effects model, compared to the control groups, there were no significant differences in the risk of diarrhea among psoriasis patients treated with these three interleukin inhibitors, and sensitivity analysis demonstrated good data robustness. Additionally, no cases of diarrhea were reported in the Ixekizumab group and Ustekinumab group.

**Conclusions:**

There is insufficient evidence to confirm that Ustekinumab, Bimekizumab, Secukinumab, and Brodalumab significantly increase the risk of new-onset IBD. However, compared to the control group, Ixekizumab was significantly associated with an increased risk of new-onset IBD in psoriasis patients. Psoriasis patients receiving Ixekizumab treatment should remain vigilant for gastrointestinal symptoms, particularly in high-risk patients, to identify and manage potential IBD early. Additionally, compared to the control group, no significant difference was observed in the risk of diarrhea as an adverse event among patients treated with Bimekizumab, Secukinumab, and Brodalumab.

**Systematic Review Registration:**

https://www.crd.york.ac.uk/PROSPERO/view/CRD42024608423, identifier CRD42024608423.

## 1 Introduction

Psoriasis is a chronic, recurrent, inflammatory, and systemic immune-mediated disease triggered by the interaction between genetic and environmental factors. Its typical clinical manifestations include scaly erythema or plaques, which may be localized or widespread. The etiology of psoriasis involves various factors, including genetics, immunity, and environmental influences (1). The global prevalence of psoriasis among adults ranges from 0.51% to 11.43%, with the highest prevalence in children at 1.37% (2, 3). Currently, biologic agents have been chosen as the primary systemic treatment for psoriasis, playing a significant and effective role in managing severe, refractory, and special types of psoriasis (4). In addition, biologics are employed as either standalone treatments or in conjunction with other systemic or topical medications for psoriasis management, according to the psoriasis management guidelines of the dermatology societies in Singapore, the United States, and Canada (5–7). Interleukin (IL) inhibitors are commonly used biological agents for the treatment of psoriasis, including the IL-12/23 (P40 subunit) inhibitor Ustekinumab, the IL-23 (P19 subunit) inhibitor Guselkumab, and IL-17A inhibitors such as Secukinumab, Ixekizumab, Bimekizumab, Brodalumab, and others. IBD is a chronic inflammatory condition with a prevalence of approximately 0.3%, affecting the small intestine, colon, and rectum. It includes two subtypes: Ulcerative colitis (UC) and Crohn’s disease (CD) (8). There has been demonstrated to be a higher expression of IL-17 in the intestinal mucosa of patients with CD and UC (9), but it has been found that anti-IL-17 agents fail to show clinical efficacy and may cause exacerbations of symptoms (10–12) and clinical relapses in patients with IBD (13, 14). On the other hand, there had been a central role played by IL-23 regulation in the pathogenesis of IBD, with there being a key link in the disease process. It had been suggested by studies that there was a promising therapeutic target in IL-23 for suppressing intestinal inflammation (15). However, the study indicated that IL-23 is produced in response to microbial colonization, and blocking IL-23 may disrupt the microbiota composition, increasing susceptibility to intestinal infections and exacerbate intestinal inflammation (16).

Given that it remains unclear whether the new-onset IBD in psoriasis patients is attributable to the disease itself or the treatment with interleukin inhibitors, therefore, the objective of this study is to perform a meta-analysis of RCTs to clarify the risk of new-onset IBD in psoriasis patients following treatment with five interleukin inhibitors(including Bimekizumab, Ixekizumab, Secukinumab, Brodalumab, and Ustekinumab), as well as the differences between them.

## 2 Materials and methods

### 2.1 Data sources, search strategy and study selection

This article was carried out and reported by the Preferred Reporting Items for Systematic Reviews and Meta-analysis (17). The methods were stipulated in a protocol that was registered in PROSPERO (CRD42024608423). We searched PubMed, Embase, Cochrane Library, and Web of Science for observational studies published as full-text articles in English, from establishment to June 21, 2024.

The search strategy was designed and conducted by an experienced medical librarian with input from the study investigators. The studies were identified by combining three search themes, interleukin inhibitors, inflammatory bowel disease, and a combination of psoriasis, psoriasiform, dermatological, skin, and cutaneous. The detailed search strategies are available in **Supplementary Table S1**. Regarding the inclusion criteria, no limitations were imposed on age, gender, or study duration. All RCTs in English that reported the incidence of IBD in psoriasis patients treated with interleukin inhibitors were included. When duplicate publications were identified, only the article with the newest and most comprehensive information was included. The studies with insufficient data(such as those only presenting all dermatological events), meeting abstracts, case reports, editorials, reviews, or nonhuman investigations were excluded. RCTs without a valid control group were excluded from the study.

### 2.2 Data extraction and outcome assessment

All data were independently extracted by three researchers (ZJX, LWW, and HLJ) using data extraction forms, and the eligibility of the studies was assessed. Any discrepancies were resolved by other researchers (LS and QZK). Data on study characteristics were collected, including the first author, publication year, sample size, study design, study duration, incidence of IBD, incidence of diarrhea, type of psoriasis diagnosis, patient demographics, and clinical characteristics. The diagnosis and determination of IBD were made according to the criteria of each study and were not limited by reporting methods. Two published studies included data from multiple RCTs with different regimens or subject characteristics. For instance, the study by Lebwohl on interleukin inhibitors included comparisons between placebo and patients receiving Brodalumab, as well as between placebo and patients receiving Ustekinumab (18). In this study, these subgroup comparisons were treated as independent RCTs to ensure the accuracy of the comparisons and minimize the risk of selection bias. The Cochrane risk of bias tool was used to assess the risk of bias in the RCTs (19).

### 2.3 Outcome assessment

The primary outcome was the risk difference (RD) in new-onset IBD in psoriasis patients treated with interleukin inhibitors compared to the placebo or non-interleukin inhibitors. The secondary outcome was the RD of diarrhea. Data were analyzed based on the intention-to-treat principle.

### 2.4 Data synthesis and analysis

Calculations and figures were generated using RevMan 5.4 or Stata Statistical Software version 16.0. All statistical tests were performed with a two-sided α value of 0.05 for significance. A fixed-effect model was used in this study to conduct a meta-analysis of all new-onset IBD cases reported in the experimental and control groups of psoriasis patients treated with five interleukin inhibitors. Mantel-Haenszel (MH) RD was selected as the primary analysis method because it does not exclude studies with zero events in either group and allows for the inclusion of data from all studies into the meta-analysis without continuity constraints. Due to the uncertainty surrounding the preferred methods for conducting meta-analyses of studies involving rare events (20), our approach uses various meta-analysis methods and leave-one-out sensitivity analysis to further test the validity and robustness of the effect estimates. Heterogeneity across trials of each therapy was assessed by using the I^2^ statistic, which estimates the percentage of variability that can be attributed to between‐study differences. An I^2^ value of > 50% indicates considerable heterogeneity (21). A fixed-effect model was used to perform a meta-analysis of all reported diarrhea adverse events in the experimental groups treated with interleukin inhibitors and control groups of psoriasis patients. Mantel-Haenszel (MH) RD was selected as the primary analysis method, with all statistical tests using a two-sided α value of 0.05 to determine significance. Additionally, publication bias was assessed graphically using a funnel plot.

## 3 Results

### 3.1 Study selection and characteristics

**Figure 1:**
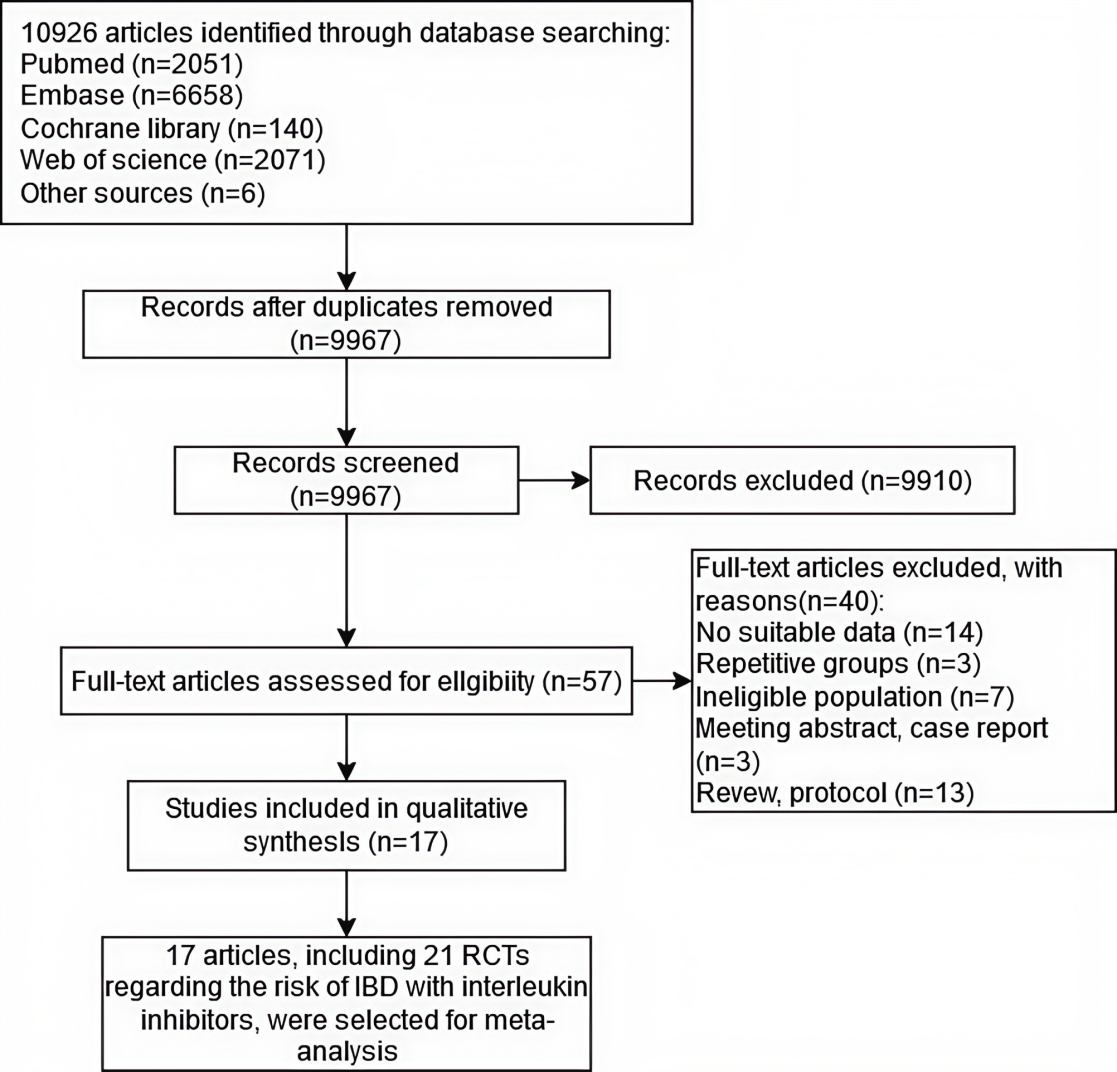
Flow chart of the assessment of the studies identified in the meta-analysis.

The search identified 10,926 studies related to interleukin inhibitors, of which 959 were removed for duplication, and 9,910 were excluded after the initial screening of titles and abstracts. In addition, 14 studies were excluded for insufficient data, 3 studies were duplicates, 7 studies did not meet the population criteria, 3 were conference abstracts and case reports, and 13 were reviews and protocols. A total of 40 studies were further excluded. A final number of 17 full‐text articles met all eligibility criteria and included 21 RCTs with a total of 12,185 patients treated with interleukin inhibitors and 4,372 treated with non-interleukin inhibitors or placebo as control (18, 22–37). The search flowchart is shown in **Figure 1**, and the detailed search strategy is provided in **Supplementary Table S1**. The 21 RCTs included seven studies of Bimekizumab (with 2,389 psoriasis patients in the experimental group and 776 in the control group), four of Ixekizumab (with 5,191 psoriasis patients in the experimental group and 1,350 in the control group), three of Secukinumab (with 843 psoriasis patients in the experimental group and 584 in the control group), three of Brodalumab (with 2,916 psoriasis patients in the experimental group and 844 in the control group), and four trials for Ustekinumab (with 846 psoriasis patients in the experimental group and 818 in the control group), respectively. The study population included patients with psoriasis and psoriatic arthritis and the study period ranged from 12 to 156 weeks. The characteristics and outcomes of the included studies are summarised in **Tables 1** and **2**. The risk of bias among RCTs is also summarised in **Supplementary Figure S1**.

**Table 1:** Characteristics of RCTs involving interleukin inhibitors.

Drug	Diseases	References	Regimen (mg)	NCT	Age Mean (SD)	Sex (male %)	Study duraiton (wk)
Bimekizumab	Psoriasis	Kristian 2021(1) (33)	320 Q4W	NCT03370133	45(14)	71	52
	psoriatic arthritis	Christopher 2023 (34)	160 Q4W	NCT03895203	48(13)	45	52
	psoriatic arthritis	Ritchlin 2020 (35)	16;160;320 Q4W	NCT02969525	49(13)	49	12
	psoriatic arthritis	Merola 2023 (29)	160 Q4	NCT03896581	50(12)	49	16
	Psoriasis	Warren 2021 (37)	320	NCT03412747	45(13)	65	24
	Psoriasis	Gordon 2021 (26)	320 Q4W	NCT03410992	45(13)	73	16
	psoriatic arthritis	Coates 2023 (23)	160 Q4W	NCT04009499	50(12)	49	52
Ixekizumab	psoriatic arthritis	Josef 2020 (36)	–	NCT03151551	48(12)	57	52
	Psoriasis	Gordon 2016 (25)	80;160	NCT01474512, NCT01597245, NCT01646177	46(13)	68	60
	psoriatic arthritis	Combe 2020 (24)	80Q2W; 80Q4W	NCT01695239, NCT02349295, NCT02584855	50(12)	46	24
	Psoriasis	Reich 2019 (32)	80;160	NCT02634801	44(14)	78	24
Secukinumab	psoriatic arthritis	McInnes 2015 (28)	75, 150, or 300 mg QW to Week 3, then Q4W from Week 4	NCT01752634	47(12)	49	16
	psoriatic arthritis	McInnes 2020 (27)	300 at 1–4 wk; after Q4	NCT02745080	49(12)	49	52
	Psoriasis	Blauvelt 2015 (22)	150;300	NCT01555125	46(14)	78	12
Brodalumab	Psoriasis	Lebwohl et al(AMAGINE-2)(1) 2015 (18)	140 Q2W; 210 Q2W	NCT01708603, NCT01708629	45(13)	68	12
	Psoriasis	Lebwohl et al(AMAGINE-3)(3) 2015 (18)	140 Q2W; 210 Q2W	NCT01708603, NCT01708629	45(13)	69	12
	Psoriasis	Papp 2016 (30)	140 Q2W; 210 Q2W	NCT01708590	46(12)	73	52
Ustekinumab	psoriatic arthritis	Rahman 2020 (31)	45; 90	NCT00741793	53(12)	37	365
	Psoriasis	Lebwohl et al(AMAGINE-2)(2) 2015 (18)	0.45 mg/kg; 0.9 mg/kg	NCT01708603, NCT01708629	45(13)	68	12
	Psoriasis	Lebwohl et al(AMAGINE-3)(4) 2015 (18)	0.45 mg/kg; 0.9 mg/kg	NCT01708603, NCT01708629	45(13)	68	12
	Psoriasis	Kristian 2021(2) (33)	45 mg/90 mg Q12	NCT03370133	46(13)	72	52

All doses are expressed in milligrams (mg); Q2W=every 2 weeks, Q4W=every 4 weeks, QW=weekly. In studies by the same author, all trial results are annotated with corresponding reference numbers in parentheses to differentiate between the various trials. Kristian(1)2021 (33), Kristian(2)2021 (33), Lebwohl(AMAGINE-2)(1)2015 (18), Lebwohl(AMAGINE-3)(3)2015 (18), Lebwohl(AMAGINE-2)(2)2015 (18), Lebwohl(AMAGINE-3)(4)2015 (18); - not described in the article.

**Table 2:** Characteristics of RCTs using interleukin inhibitors in comparison with control groups for incident IBD and diarrhea.

Drug	References		Total Number of Patients (n)		Incidence of IBD (n)		Incidence of Diarrhoea (n)	
		Diagnosis method	Trial	Placebo	Trial	Placebo	Trial	Placebo
Bimekizumab	Kristian(1) 2021 (33)	①	321	83	1	0	0	0
	Christopher 2023 (34)	①	702	140	2	0	36	7
	Ritchlin 2020 (35)	④	164	42	0	0	0	0
	Merola 2023 (29)	①	267	133	0	0	0	0
	Warren 2021 (37)	④	319	159	0	0	13	4
	Gordon 2021 (26)	③	349	86	0	0	0	0
	Coates 2023 (23)	④	267	133	0	0	0	0
Ixekizumab	Josef 2020 (36)	②	283	283	1	0	0	0
	Gordon 2016 (25)	①	3736	791	11	0	0	0
	Combe 2020 (24)	②	1118	224	2	0	0	0
	Reich 2019 (32)	④	54	52	0	0	0	0
Secukinumab	McInnes 2015 (28)	③	299	98	2	0	7	3
	McInnes 2020 (27)	④	426	427	2	0	31	35
	Blauvelt 2015 (22)	③	118	59	0	0	7	1
Brodalumab	Lebwohl (AMAGINE-2)(1) 2015 (18)	③	1222	309	1	0	0	0
	Lebwohl (AMAGINE-3)(3) 2015 (18)	③	1253	315	0	0	1	0
	Papp 2016 (30)	③	441	220	0	0	0	0
Ustekinumab	Rahman 2020 (31)	④	70	111	0	1	0	0
	Lebwohl (AMAGINE-2)(2) 2015 (18)	③	300	309	0	0	0	0
	Lebwohl (AMAGINE-3)(4) 2015 (18)	③	313	315	0	0	0	0
	Kristian(2) 2021 (33)	①	163	83	0	0	0	0

IBD, inflammatory bowel disease; ①IBD events were adjudicated by an external committee. ②IBD events were adjudicated by an external committee and verified against the EPIMAD criteria for IBD diagnosis. ③Only adverse cardiovascular events were described as being adjudicated by an external committee. ④Not specified.

### 3.2 Meta-analysis of the risk of new-onset IBD in psoriasis patients treated with interleukin inhibitors

#### 3.2.1 Bimekizumab

**Figure 2:**
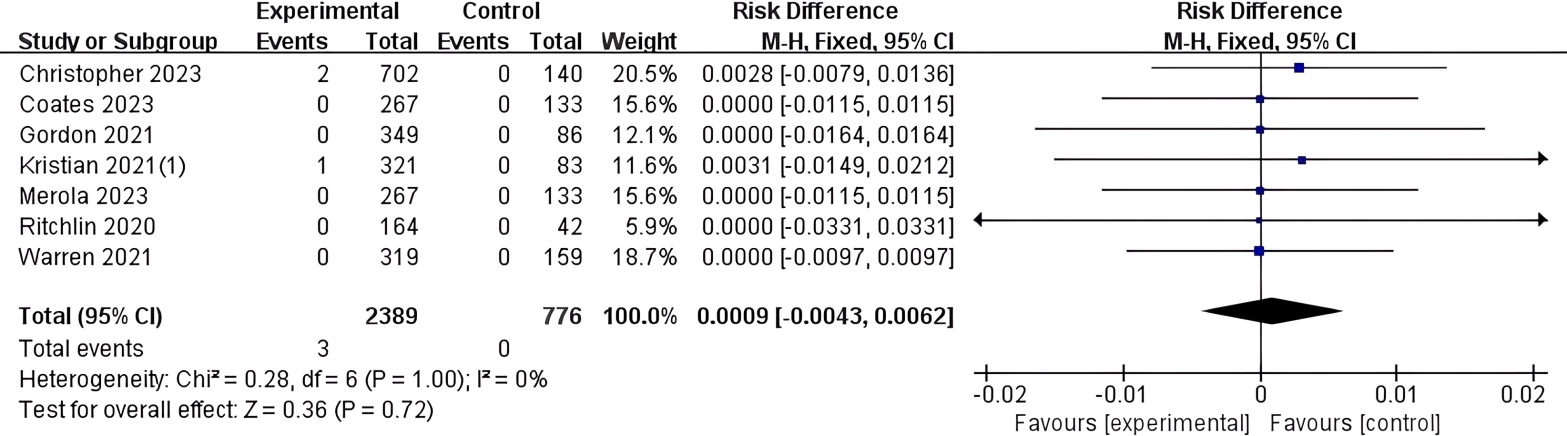
Meta-analysis of the risk difference (MH RD) for new-onset IBD comparing Bimekizumab group with the control group based on the fixed-effect model. In studies by the same author, all trial results are annotated with corresponding reference numbers in parentheses to differentiate between the various trials. Kristian(1)2021 (33).

In this study, 7 articles were included that conducted statistical analysis on the risk of IBD induction with Bimekizumab. Relevant data were extracted, and among the 2,389 patients in the experimental group, 3 cases of new-onset IBD were reported as an adverse event. No new-onset IBD cases were reported in the 776 patients in the control group. A meta-analysis was performed on the 7 included studies, and a forest plot was generated (**Figure 2**). The results showed I² = 0% and P > 0.05, indicating no heterogeneity among the studies included. Therefore, a meta-analysis based on a fixed-effect model showed that the overall risk of new-onset IBD with Bimekizumab was not statistically different compared to the control (MH RD 0.0009, 95% CI -0.0043-0.0062, I² = 0%, P = 0.72).

#### 3.2.2 Ixekizumab

**Figure 3:**
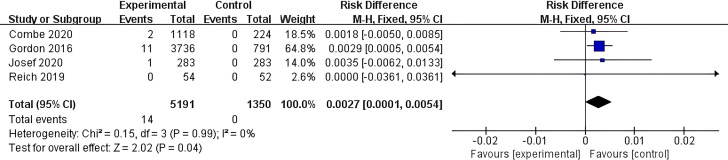
Meta-analysis of the risk difference (MH RD) for new-onset IBD comparing Ixekizumab group with the control group based on the fixed-effect model.

Four articles were included in this study that conducted statistical analysis on the risk of new-onset IBD induction with Ixekizumab. Relevant data were extracted, and among the 5,191 patients in the experimental group, 14 cases of new-onset IBD were reported as an adverse event. No new-onset IBD cases were reported in the 1,350 patients in the control group. Meta-analysis was performed on the 4 included studies, and a forest plot was generated (**Figure 3**). The results showed I² = 0% and P < 0.05, indicating no heterogeneity among the 4 studies included. Therefore, a fixed-effect model was used for the meta-analysis. The results revealed that compared to the control group, the overall risk of new-onset IBD with Ixekizumab was statistically different (MH RD 0.0027, 95% CI 0.0001 - 0.0054, I² = 0%, P = 0.04).

#### 3.2.3 Secukinumab

**Figure 4:**
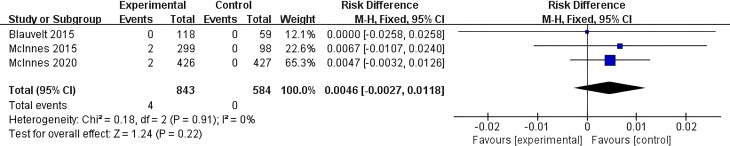
Meta-analysis of the risk difference (MH RD) for new-onset IBD comparing Secukinumab group with the control group based on the fixed-effect model.

In this study, 3 articles were included that conducted statistical analysis on the risk of IBD induction with Secukinumab. Relevant data were extracted, and among the 843 patients in the experimental group, 4 cases of new-onset IBD were reported as an adverse event. No new-onset IBD cases were reported in the 584 patients in the control group. A meta-analysis was performed on the 3 included studies, and a forest plot was generated (**Figure 4**). The results showed I² = 0% and P > 0.05, indicating no heterogeneity among the 3 studies included. Therefore, a fixed-effect model was used for the meta-analysis. The results revealed that compared to the control group, the overall risk of new-onset IBD with Secukinumab was not significantly different (MH RD 0.0046, 95% CI -0.0027-0.0118, I^2^ = 0%, P=0.22).

#### 3.2.4 Brodalumab

**Figure 5:**
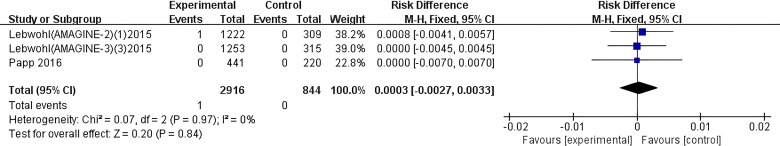
Meta-analysis of the risk difference (MH RD) for new-onset IBD comparing Brodalumab group with the control group based on the fixed-effect model. In studies by the same author, all trial results are annotated with corresponding reference numbers in parentheses to differentiate between the various trials. Lebwohl(AMAGINE-2)(1)2015 (18), Lebwohl(AMAGINE-3)(3)2015 (18).

In this study, 3 articles were included that conducted statistical analysis on the risk of IBD induction with Brodalumab. Relevant data were extracted, and among the 2,916 patients in the experimental group, 1 case of new-onset IBD was reported as an adverse event. No new-onset IBD cases were reported in the 844 patients in the control group. A meta-analysis was performed on the 3 included studies, and a forest plot was generated (**Figure 5**). The results showed I² = 0% and P > 0.05, indicating no heterogeneity among the 3 studies included. Therefore, a fixed-effect model was used for the meta-analysis. The results revealed that compared to the control group, the overall risk of new-onset IBD with Brodalumab was not significantly different (MH RD 0.0003, 95% CI -0.0027-0.0033, I^2^ = 0%, P=0.84).

#### 3.2.5 Ustekinumab

**Figure 6:**
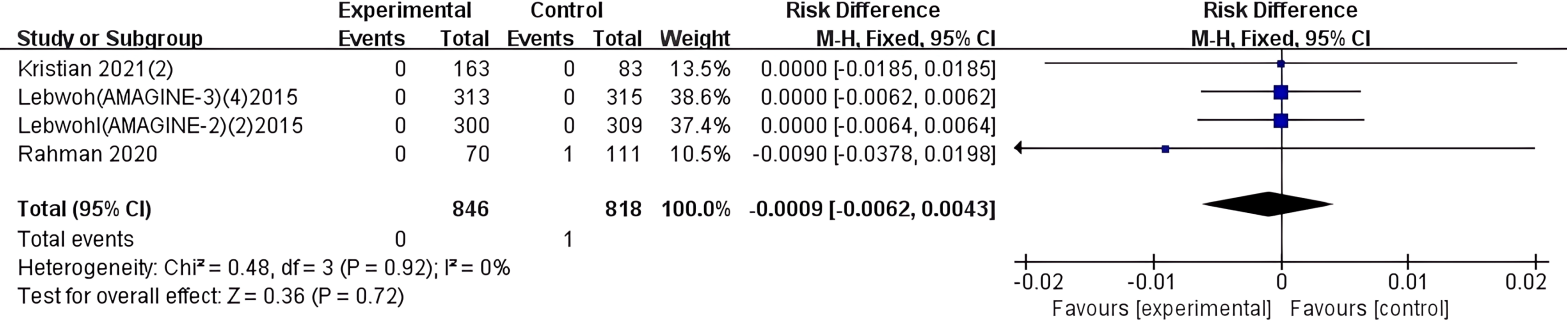
Meta-analysis of the risk difference (MH RD) for new-onset IBD comparing Ustekinumab group with the control group based on the fixed-effect model. In studies by the same author, all trial results are annotated with corresponding reference numbers in parentheses to clearly differentiate between the various trials. Kristian 2021(2) (33), Lebwohl(AMAGINE-2)(2)2015 (18), Lebwohl(AMAGINE-3)(4)2015 (18).

In this study, 4 articles were included that conducted statistical analysis on the risk of IBD induction with Ustekinumab. Relevant data were extracted, and among the 818 patients in the control group, 1 case of new-onset IBD was reported as an adverse event. No new-onset IBD cases were reported in the 846 patients in the experimental group. A meta-analysis was performed on the 4 included studies, and a forest plot was generated (**Figure 6**). The results showed I² = 0% and P > 0.05, indicating no heterogeneity among the 4 studies included. Therefore, a fixed-effect model was used for the meta-analysis. The results revealed that compared to the control group, the overall risk of new-onset IBD with Ustekinumab was not significantly different (MH RD -0.0009, 95% CI -0.0062-0.0043, I^2^ = 0%, P=0.72).

#### 3.2.6 Summary of risk of new-onset IBD across interleukin inhibitors

Among 12,185 patients in the experimental group of the 21 RCTs, 22 new-onset cases of IBD were reported as adverse events, including 13 cases diagnosed with UC, 7 cases diagnosed with CD, and 2 cases without a specified type described in the articles. In the control group of 4,372 patients, 1 new-onset case of IBD was reported as an adverse event, with no specific type described in the article. Among these cases, 3 were associated with Bimekizumab, 14 with Ixekizumab, 4 with Secukinumab, 1 with Brodalumab, and 0 with Ustekinumab. Corresponding to an incidence of 2.4 cases per 1000 patient-years among those treated with interleukin inhibitors. Specifically, the annual incidence was 1.8 cases per 1000 patient-years for Bimekizumab, 2.7 cases per 1000 patient-years for Ixekizumab, 7.3 cases per 1000 patient-years for Secukinumab, and 0.98 cases per 1000 patient-years for Brodalumab. No cases were reported for Ustekinumab. Detailed data can be found in **Supplementary Table S2**. Furthermore, we conducted statistical analyses on the risk of new-onset IBD for all included interleukin inhibitors and generated corresponding forest plots (**Supplementary Figure S2**). The results showed I² = 0% and P > 0.05, indicating no heterogeneity among the 21 studies included. Therefore, a fixed-effect model was used for the meta-analysis. The results revealed that compared to the control group, the overall risk of new-onset IBD with IL inhibitors was not significantly different (MH RD 0.0016, 95% CI -0.0003-0.0034, I^2^ = 0%, P=0.10). While consistency metrics suggest homogeneous effects, the observed I²=0% may reflect limited power to detect heterogeneity due to the few included studies and rare events. Clinical interpretation should consider possible undetected variability in real-world populations (38, 39). In summary, the use of Bimekizumab, Secukinumab, Brodalumab, and Ustekinumab for the treatment of psoriasis showed no significant difference in the incidence of new-onset IBD compared to the control group. However, Ixekizumab was significantly associated with an increased risk of IBD compared to the control group, suggesting it may elevate the risk of IBD development. Funnel plot analysis revealed no substantial asymmetry, indicating no significant publication bias. The funnel plot is presented in **Supplementary Figure S3**. Although the risk difference for new-onset IBD with secukinumab was not statistically significant (MH RD 0.0046, 95% CI -0.0027-0.0118; Z=1.24, P=0.22), its point estimate was comparable to that of ixekizumab. Given secukinumab’s smaller sample size (n=843 vs. ixekizumab n=5,191), the current analysis may have been underpowered to detect a potentially significant association. Thus, despite the lack of statistical significance, clinicians should remain vigilant for IBD symptoms in patients receiving IL-17 inhibitors, particularly those with predisposing factors. Larger-scale studies are warranted to clarify secukinumab’s IBD risk profile.

### 3.3 Meta-analysis of diarrhea adverse events in psoriasis patients treated with three interleukin inhibitors

#### 3.3.1 Bimekizumab

**Figure 7:**
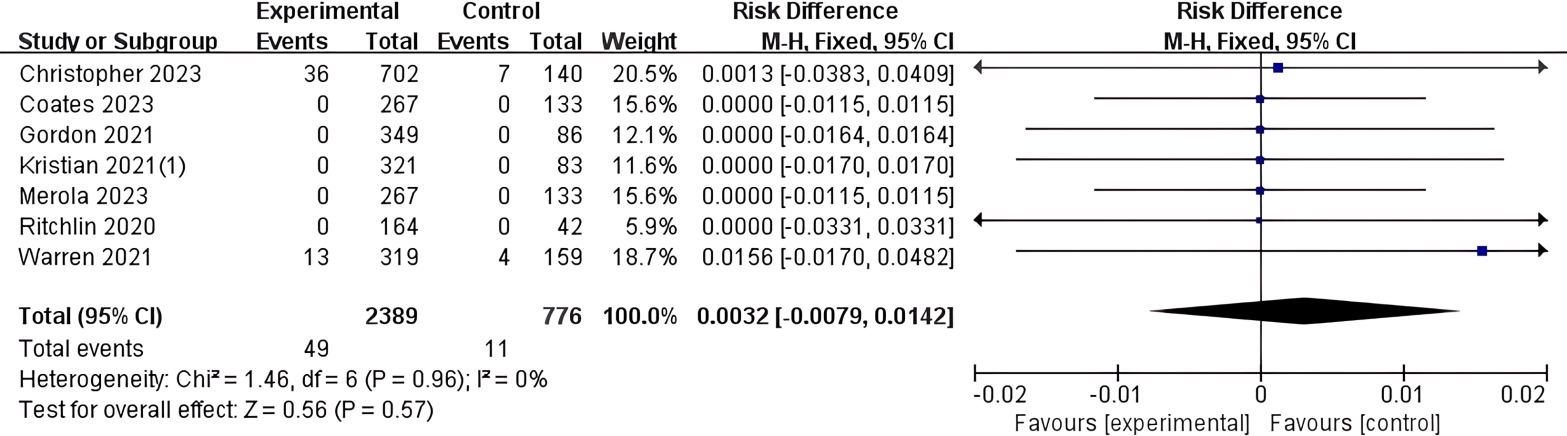
Meta-analysis of the risk difference (MH RD) for diarrhea adverse events comparing Bimekizumab group with the control group based on the fixed-effect model. In studies by the same author, all trial results are annotated with corresponding reference numbers in parentheses to clearly differentiate between the various trials. Kristian 2021(1) (33).

In this study, 7 articles were included that provided statistical analyses on the risk of diarrhea as an adverse event associated with Bimekizumab. Relevant data were extracted, revealing that among 2,389 patients in the experimental group, 49 cases reported diarrhea as an adverse event, while among 776 patients in the control group, 11 cases reported diarrhea as adverse events. A meta-analysis was performed on the 7 included studies, and a forest plot was generated (**Figure 7**). The results showed I² = 0% and P > 0.05, indicating no heterogeneity among the 7 studies. Therefore, meta-analysis based on a fixed-effect model showed that the overall risk of diarrhea in Bimekizumab group was not statistically different compared to the control group (MH RD 0.0032, 95% CI -0.0079-0.0142, I^2^ = 0%, P=0.57).

#### 3.3.2 Secukinumab

**Figure 8:**
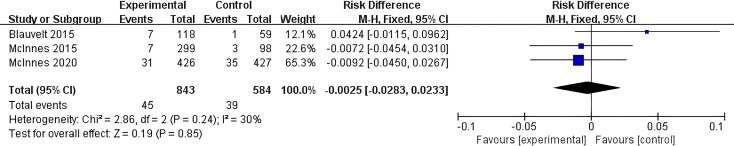
Meta-analysis of the risk difference (MH RD) for diarrhea adverse events comparing Secukinumab group with the control group based on the fixed-effect model.

In this study, 3 included articles provided statistical analyses on the risk of diarrhea as an adverse event associated with Secukinumab. Relevant data were extracted, revealing that among 843 patients in the experimental group, 45 cases reported diarrhea as an adverse event, while among 584 patients in the control group, 39 cases reported diarrhea as an adverse event. A meta-analysis was performed on the 3 included studies, and a forest plot was generated (**Figure 8**). The results showed I² = 30% and P > 0.05, indicating low heterogeneity among the three included studies. Therefore, a meta-analysis based on a fixed-effect model showed that the overall risk of diarrhea in Secukinumab group was not statistically different compared to the control group (MH RD -0.0025, 95% CI -0.0283-0.0233, I^2^ = 30%, P=0.85).

#### 3.3.3 Brodalumab

**Figure 9:**
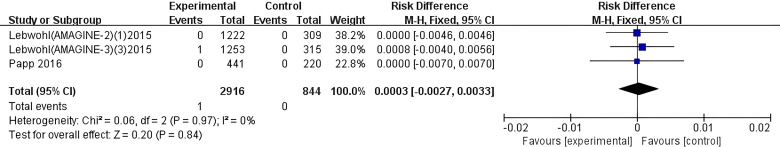
Meta-analysis of the risk difference (MH RD) for diarrhea adverse events comparing Brodalumab with the control group based on the fixed-effect model. In studies by the same author, all trial results are annotated with corresponding reference numbers in parentheses to clearly differentiate between the various trials. Lebwohl(AMAGINE-2)(1)2015 (18), Lebwohl(AMAGINE-3)(3)2015 (18).

Three RCTs were included in this study to analyze the risk of diarrhea as an adverse event associated with Brodalumab. Data extraction showed that among 2,916 patients in the experimental group, 1 case was reported as a diarrhea adverse event, while no cases were reported in the 844 patients in the control group. A meta-analysis was performed on the 3 included studies, and a forest plot was generated (**Figure 9**). The results showed I² = 0% and P > 0.05, indicating no heterogeneity among the 3 studies included. Therefore, meta-analysis based on a fixed-effect model showed that overall risk of diarrhea in Brodalumab group was not statistically different compared to control group (MH RD 0.0003, 95% CI -0.0027-0.0033, I^2^ = 0%, P=0.84).

#### 3.3.4 Summary of diarrhea adverse events across interleukin inhibitors

Since no diarrhea-related adverse events were reported in the RCTs of Ixekizumab and Ustekinumab, only 13 RCTs related to Bimekizumab, Secukinumab, and Brodalumab were included in the analysis of diarrhea. Among the 6,148 patients in the experimental group, 95 cases of diarrhea were reported as adverse events, while 50 cases were reported in the 2,204 patients in the control group. Additionally, no diarrhea-related adverse events were reported for Ixekizumab or Ustekinumab. Furthermore, we conducted statistical analyses on the risk of diarrhea for all included interleukin inhibitors and generated corresponding forest plots (**Supplementary Figure S4**). The results showed I² = 0% and P > 0.05, indicating no heterogeneity among the 13 studies included. Therefore, a fixed-effect model was used for the meta-analysis. The results revealed that compared to the control group, the overall risk of diarrhea with IL inhibitors was not significantly different (MH RD 0.0008, 95% CI -0.0062-0.0077, I^2^ = 0%, P=0.83). In summary, the incidence of diarrhea as an adverse event in psoriasis patients treated with the three interleukin inhibitors showed no significant difference compared to the control group. Funnel plot analysis revealed no substantial asymmetry, indicating no significant publication bias. The funnel plot is presented in **Supplementary Figure S5**.

### 3.4 Sensitivity analyses

A meta-analysis of the 4 included RCTs investigating Ixekizumab was performed using both fixed-effect and random-effects models (24, 25, 32, 36). The results were(MH RD 0.027, 95% CI 0.0001-0.0054, I^2^ = 0%, P = 0.04) for the fixed-effect model and (MH RD 0.0028, 95% CI 0.0006-0.0051, I^2^ = 0%, P=0.01) for the random-effects model, showing no substantial change due to the model selection. Sensitivity analysis, conducted by sequentially excluding individual studies, revealed that the risk difference (RD) for new-onset IBD in psoriasis patients treated with Ixekizumab ranged from 0.0023 to 0.0030, with a 95% CI ranging from -0.0032 to 0.0078. The results indicated that after excluding the RCT published by Gordon (25), the pooled analysis yielded an MH RD of 0.0023 (95% CI: -0.0032 to 0.0078, I² = 0%, P = 0.41), suggesting that this study has a significant impact on the overall results. The data revealed that the excluded RCT included a large number of patients in the experimental group and reported a higher number of new-onset IBD cases. The analysis results based on the random-effects model are illustrated in **Supplementary Figure S6**.

## 4 Discussion

This systematic review and meta-analysis integrated data from 17 RCTs, encompassing a total of 16,557 psoriasis patients, to evaluate and compare the risk of new-onset IBD in patients treated with five distinct interleukin inhibitors, thereby providing scientific evidence for clinical practice. The research findings indicated that although the use of interleukin inhibitors was associated with a risk of new-onset IBD, the overall risk remained low. In most cases, the administration of these drugs did not significantly increase the incidence of IBD. The meta-analysis revealed no significant differences in the risk of new-onset IBD between Bimekizumab, Secukinumab, Brodalumab, Ustekinumab group and their control groups. However, compared to the control group, Ixekizumab was significantly associated with an increased risk of new-onset IBD in psoriasis patients (MH RD 0.0027, 95% CI 0.0001-0.0054, I² = 0%, P = 0.04). Compared with the meta-analysis by Yamada et al. (40), our study identified a significant association between Ixekizumab and the risk of new-onset IBD—an observation that was not clearly emphasized in earlier analyses. First, given the low heterogeneity across studies and the rarity of new-onset IBD events in psoriasis patients treated with interleukin inhibitors, we adopted a fixed-effects model combined with the Mantel-Haenszel method to estimate risk differences. This approach enhances the clinical interpretability of absolute risk estimates, mitigates the potential overestimation associated with odds ratios in sparse-event settings, and allows for the inclusion of studies reporting zero events. Second, by incorporating recently published randomized controlled trials and expanding the overall sample size, our study improves the precision of statistical estimates and the reliability of the findings.

Multiple case reports and clinical studies support the findings of this research, indicating that psoriasis patients may be associated with developing new-onset IBD following treatment with Ixekizumab (41, 42). However, reports of new-onset IBD associated with Secukinumab, Bimekizumab, Brodalumab, and Ustekinumab were relatively rare. Notably, existing research suggested that Ustekinumab is not only effective in treating IBD but can also alleviate new-onset IBD induced by Ixekizumab (43, 44). Schreiber analyzed data from 21 clinical trials and found that the exposure-adjusted incidence rates of new-onset IBD among psoriasis patients treated with Secukinumab ranged between 0.01 and 0.13, indicating that such events were uncommon (45). Furthermore, Wang conducted data mining and analysis on interleukin-17A inhibitor-associated IBD adverse events based on the FAERS database and noted that Ixekizumab is associated with a risk of new-onset IBD (46). IL-17 is a crucial inflammatory cytokine in the Th17/IL-23 pathway and is highly expressed in the intestinal mucosa of patients with UC and CD. Although the interleukin-17 inhibitor Ixekizumab is considered to have therapeutic potential for IBD, its critical role in maintaining epithelial barrier integrity and gut microbiota balance may explain the risk of IBD development during its treatment. In contrast, interleukin-23 inhibitors (such as Ustekinumab) selectively reduce IL-17 produced through the IL-23-dependent pathway, thereby alleviating inflammation and achieving remission in IBD, while IL-23-independent IL-17 remains capable of maintaining epithelial barrier integrity and preserving microbial balance (47). It is worth noting that a previous study by Yamada et al (40). explored the association between IL-17 inhibitors and the risk of new-onset IBD, reporting no significant increase in IBD incidence associated with IL-17 inhibitors. Compared with that study, we incorporated more recent RCTs, thereby expanding the temporal scope and data coverage and enhancing the currency and representativeness of the findings. Moreover, our study not only examined three IL-17 inhibitors (Ixekizumab, Secukinumab, and Brodalumab), but also included an IL-12/23 inhibitor (Ustekinumab) and a dual IL-17A/IL-17F inhibitor (Bimekizumab), enabling a more comprehensive comparative analysis across different types of IL inhibitors. Notably, we observed a statistically significant association between Ixekizumab use and an increased risk of new-onset IBD in patients with psoriasis compared with the control group—an observation not clearly identified in previous studies (40). In addition, we included diarrhea as a secondary endpoint, considering it a potential early symptom of IBD, thereby complementing the understanding of gastrointestinal adverse events associated with interleukin inhibitors. Overall, while our results are consistent with prior studies in suggesting a low incidence of new-onset IBD among psoriasis patients treated with IL inhibitors, this study contributes novel insights by updating the evidence base, broadening the range of therapeutic agents evaluated, and incorporating additional clinically relevant outcomes. These findings provide more comprehensive evidence to support clinical decision-making in the management of psoriasis with IL inhibitors.

This meta-analysis of RCTs included 16,557 psoriasis patients from 21 published trials, allowing for risk estimation. Overall, among 12,185 psoriasis patients treated with interleukin inhibitors, 22 new-onset IBD cases were reported as adverse events, compared to 1 case among 4,372 controls. The incidence rate of new-onset IBD in patients treated with interleukin inhibitors was 2.4 per 1,000 patient-years. The low incidence rate reported in the current meta-analysis is consistent with a rate of 2.4 per 1,000 patient-years, as reported in a previous meta-analysis that investigated the risk of IBD associated with interleukin-17A inhibitors in RCTs (40). The low incidence rate reported in the current meta-analysis is consistent with the findings of a 5-year safety study on Ixekizumab in patients with psoriasis, which reported an IBD incidence rate of 0.2 per 100 patient-years, including both UC and CD (48). A similar incidence rate was also observed in a longitudinal observational study of Secukinumab in patients with plaque psoriasis (49). The incidence rate of 2.4 cases per 1000 patient-years observed in our study falls within the range reported in observational studies assessing IBD risk in psoriasis patients. However, this rate appears significantly higher compared to the incidence rate of 0.109 cases per 1000 person-years reported in the general US population (50).

Diarrhea is one of the common clinical symptoms of CD and UC. As the diagnosis of IBD is primarily confirmed through colonoscopy, patients with mild symptoms may not undergo the procedure, resulting in them remaining undiagnosed. However, most studies included in this analysis reported diarrhea as an adverse event. Since diarrhea was not reported as an adverse event in RCTs involving Ixekizumab and Ustekinumab, this study focused on 13 RCTs related to Bimekizumab (23, 26, 29, 33–35, 37), Secukinumab (22, 27, 28), and Brodalumab (18, 30). A fixed-effect model analysis revealed no significant increase in the risk of diarrhea among psoriasis patients treated with these three interleukin inhibitors. Notably, interleukin inhibitors reshape the gut microbiota through immunoregulation and microenvironmental remodeling. IL-17 and IL-23 play critical roles in structuring the intestinal microbial ecosystem and maintaining immune homeostasis. Inhibition of these cytokines may diminish pro-inflammatory microbes, compromise antimicrobial defenses, and disrupt epithelial integrity, together promoting dysbiosis and contributing to the gastrointestinal adverse effects observed during treatment (51). Further studies are needed to clarify the causal links between microbiota alterations and gastrointestinal outcomes.

In the sensitivity analysis, we employed both random-effects models and a leave-one-out approach to assess the risk of new-onset IBD in psoriasis patients treated with Ixekizumab compared to the control group, further validating the robustness of these findings. The results demonstrated that the data remained consistent and robust.

Although this study is drawn from data from a substantial number of RCTs, several limitations should be acknowledged. Despite the relatively large sample size, the rarity of IBD as a complication and the reporting of zero events in some studies may introduce bias into the statistical results. While this study provides preliminary evidence regarding the association between five interleukin inhibitors and the risk of new-onset IBD, further prospective studies and long-term follow-up researches are necessary to confirm the safety of these drugs in psoriasis patients, particularly their long-term effects on intestinal health. Although a fixed-effect model was employed in this study, significant heterogeneity may still exist due to variations in study designs and patient populations. Factors such as the type of psoriasis and study duration could influence IBD risk, yet these variables were not adequately controlled for in the included studies.

## 5 Conclusion

In summary, no significant difference in the risk of new-onset IBD was observed between the Bimekizumab, Secukinumab, Brodalumab, Ustekinumab group and control group(placebo or non-interleukin inhibitors) for the treatment of psoriasis patients. A significant difference in the risk of new-onset IBD was observed between the Ixekizumab group and the control group in the treatment of psoriasis patients(MH RD 0.0027, 95% Cl 0.0001-0.0054, I^2^ = 0%, P=0.04). Therefore, psoriasis patients undergoing Ixekizumab treatment should remain vigilant regarding gastrointestinal symptoms, particularly in high-risk individuals, to facilitate early identification and management of potential IBD.

## Data Availability

The original contributions presented in the study are included in the article/**Supplementary Material**. Further inquiries can be directed to the corresponding authors.

## Supplementary material

Supplementary Figure 1 Risk of bias assessment of studies

Supplementary Figure 2 Meta-analysis of the risk difference (MH RD) for new-onset IBD comparing Interleukin Inhibitors with the control group based on the fixed-effect model

Supplementary Figure 3 Funnel Plot for Publication Bias in the Risk of New-Onset IBD among Psoriasis Patients Treated with Interleukin Inhibitors. **(A)** Bimekizumab, **(B)** Ixekizumab, **(C)** Secukinumab, **(D)** Brodalumab, **(E)** Ustekinumab

Supplementary Figure 4 Meta-analysis of the risk difference (MH RD) for Diarrhea comparing Interleukin Inhibitors with the control group based on the fixed-effect model

Supplementary Figure 5 Funnel Plot for Publication Bias in Diarrhea Adverse Events among Psoriasis Patients Treated with Interleukin Inhibitors. **(A)** Bimekizumab, **(B)** Secukinumab, **(C)** Brodalumab

Supplementary Figure 6 Meta-analysis of the risk difference (MH RD) for new-onset IBD comparing Ixekizumab with the control group based on the random-effect model
