# Supplementary figures and images for: Risk of new-onset inflammatory bowel disease in psoriasis patients treated with five different interleukin inhibitors: a systematic review and meta-analysis
Source: Front Immunol. 2025 Jun 4;16:1594998. doi: 10.3389/fimmu.2025.1594998 (PMC12174387; doi:10.3389/fimmu.2025.1594998)

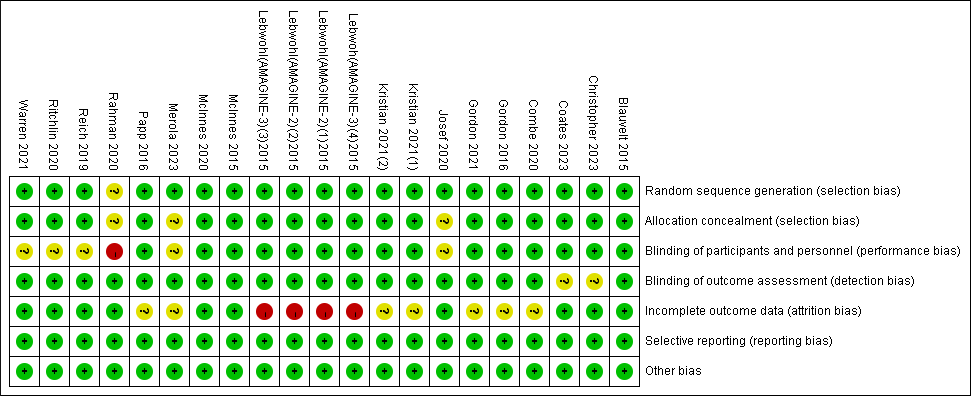

Supplement: Supplementary Figure 1 — Risk of bias assessment of studies [file Image1.png]

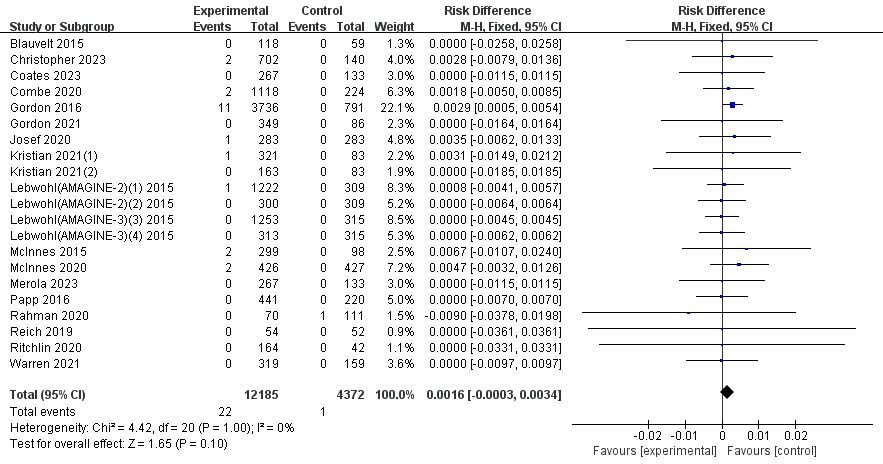

Supplement: Supplementary Figure 2 — Meta-analysis of the risk difference (MH RD) for new-onset IBD comparing Interleukin Inhibitors with the control group based on the fixed-effect model [file Image2.png]

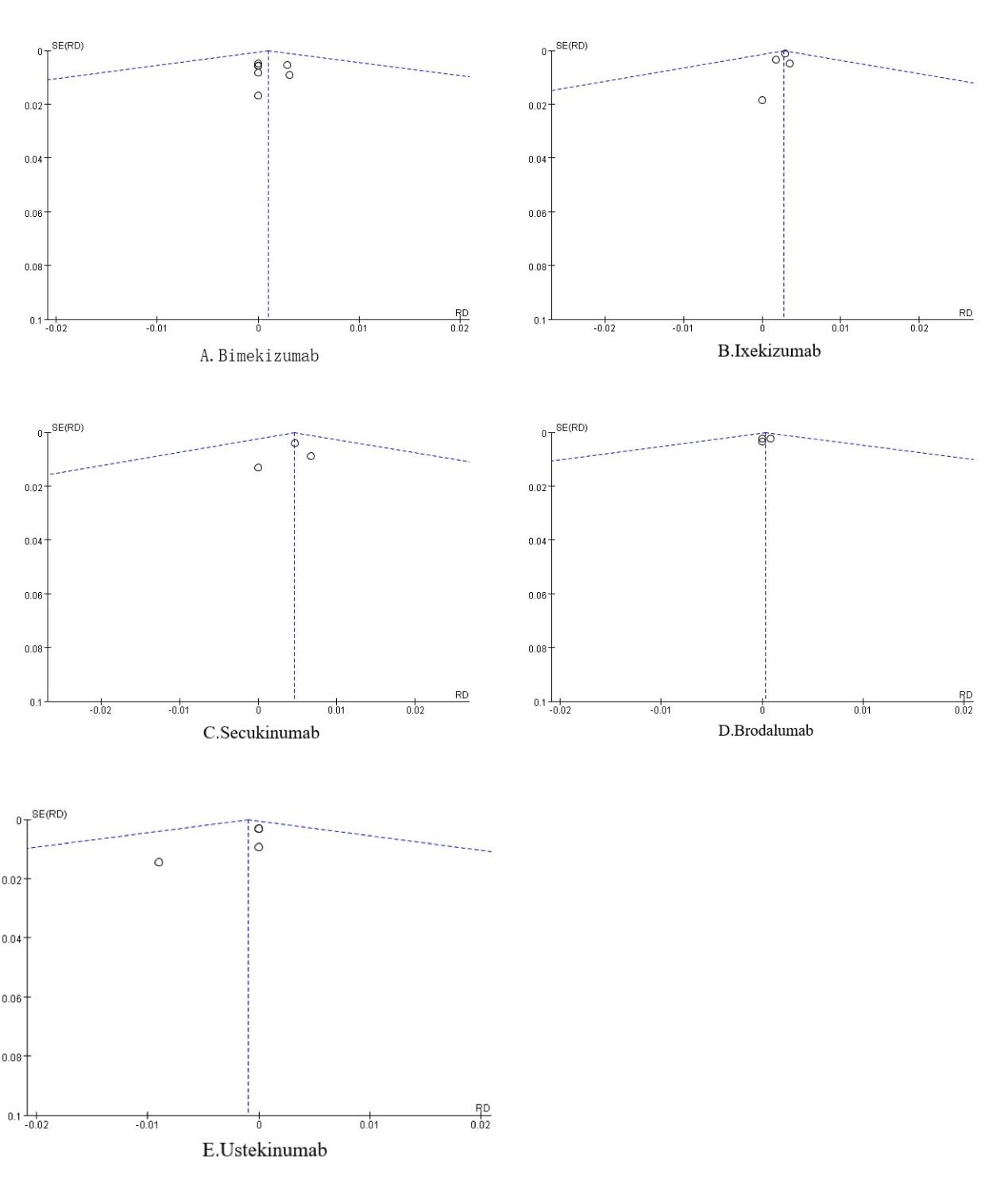

Supplement: Supplementary Figure 3 — Funnel Plot for Publication Bias in the Risk of New-Onset IBD among Psoriasis Patients Treated with Interleukin Inhibitors. (A) Bimekizumab, (B) Ixekizumab, (C) Secukinumab, (D) Brodalumab, (E) Ustekinumab [file Image3.png]

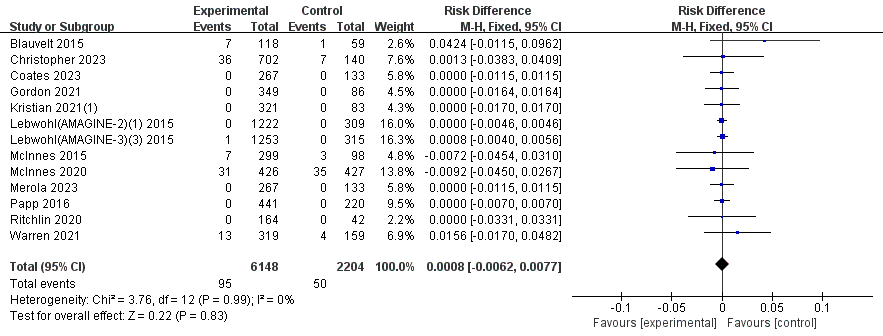

Supplement: Supplementary Figure 4 — Meta-analysis of the risk difference (MH RD) for Diarrhea comparing Interleukin Inhibitors with the control group based on the fixed-effect model [file Image4.png]

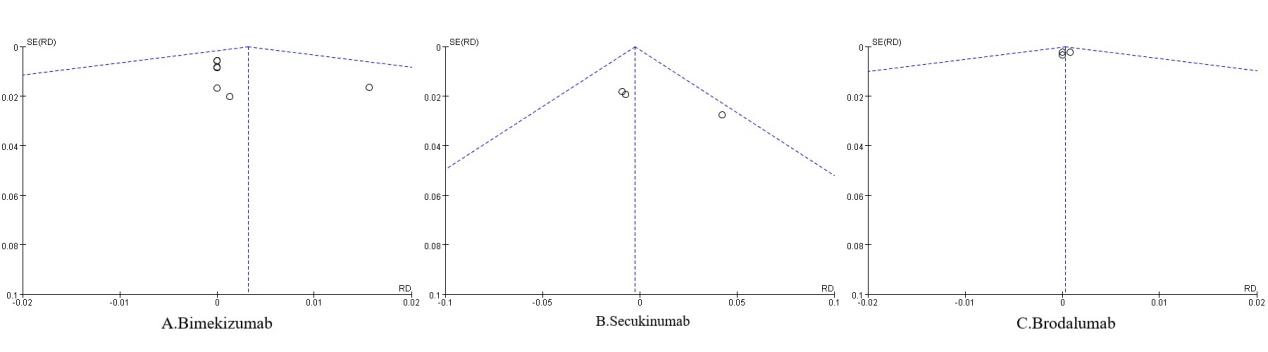

Supplement: Supplementary Figure 5 — Funnel Plot for Publication Bias in Diarrhea Adverse Events among Psoriasis Patients Treated with Interleukin Inhibitors. (A) Bimekizumab, (B) Secukinumab, (C) Brodalumab [file Image5.png]

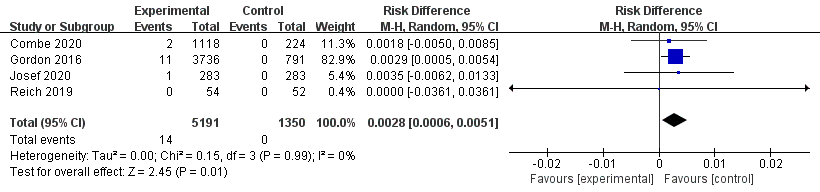

Supplement: Supplementary Figure 6 — Meta-analysis of the risk difference (MH RD) for new-onset IBD comparing Ixekizumab with the control group based on the random-effect model [file Image6.png]
