# Supplementary material for: Risk of new-onset inflammatory bowel disease in psoriasis patients treated with five different interleukin inhibitors: a systematic review and meta-analysis
Source: Front Immunol. 2025 Jun 4;16:1594998. doi: 10.3389/fimmu.2025.1594998 (PMC12174387; doi:10.3389/fimmu.2025.1594998)
Supplement: Supplementary file 7 [file Table1.docx]

**Supplemental Table 1. Search strategy:** Taking PubMed as an example

| Search number | Query | Results |
| --- | --- | --- |
| 4 | #1 and #2 and #3 | 2,051 |
| 3 | ((((psoriasis[All Fields]) OR (psoriasiform[All Fields])) OR (dermatological[All Fields])) OR (skin[All Fields])) OR (cutaneous[All Fields]) | 1,348,428 |
| 2 | ((((((((((((((((((((((((((((((((Interleukin inhibitor[All Fields]) OR (IL inhibitors[All Fields])) OR (IL[All Fields])) OR (IL inhibitor[All Fields])) OR (Interleukin antagonist[All Fields])) OR (IL antagonist[All Fields])) OR (Interleukin blocker[All Fields])) OR (Interleukin receptor blockers[All Fields])) OR (Interleukin suppressor*[All Fields])) OR (IL receptor antagonists[All Fields])) OR (Anti-interleukin therapy[All Fields])) OR (ixekizumab[All Fields])) OR (LY2439821[All Fields])) OR (Taltz[All Fields])) OR (secukinumab[All Fields])) OR (AIN 457[All Fields])) OR (Cosentyx[All Fields])) OR (guselkumab[All Fields])) OR (Tremfya[All Fields])) OR (CNTO 1959[All Fields])) OR (tildrakizumab[All Fields])) OR (SCH 900222[All Fields])) OR (MK-3222[All Fields])) OR (Ilumya[All Fields])) OR (Ustekinumab[All Fields])) OR (CNTO 1275[All Fields])) OR (Stelara[All Fields])) OR (Brodalumab[All Fields])) OR (AMG 827[All Fields])) OR (Siliq[All Fields])) OR (KHK4827[All Fields])) OR (Bimekizumab[All Fields])) OR (UCB4940[All Fields]) | 770,231 |
| 1 | (((((((((((((Inflammatory Bowel Disease[All Fields]) OR (Crohn's Disease[All Fields])) OR (Crohns Disease[All Fields])) OR (Crohn's Enteritis[All Fields])) OR (Regional Enteritis[All Fields])) OR (ileocolitis[All Fields])) OR (ileitis[All Fields])) OR (Terminal ileitis[All Fields])) OR (Regional ileitis[All Fields])) OR (Granulomatous Enteritis[All Fields])) OR (Colitis Granulomatous[All Fields])) OR (Colitis Gravis[All Fields])) OR (idiopathic Proctocolitis[All Fields])) OR (Ulcerative Colitis[All Fields]) | 153,287 |
