# Supplementary material for: Risk of new-onset inflammatory bowel disease in psoriasis patients treated with five different interleukin inhibitors: a systematic review and meta-analysis
Source: Front Immunol. 2025 Jun 4;16:1594998. doi: 10.3389/fimmu.2025.1594998 (PMC12174387; doi:10.3389/fimmu.2025.1594998)
Supplement: Supplementary file 8 [file Table2.docx]

**Supplementary Table 2.** Summary of findings

|  | Number of participants (RCTs) | Study Event Rates (%) | | Risk Difference | Anticipated absolute effects | |
| --- | --- | --- | --- | --- | --- | --- |
|  |  | Trial | Control |  | Trial | Control |
| Bimekizumab | 3165(7 RCTs) | 3/2,389 (0.125%) | 0/776 | MH RD 0.0009, (95% Cl -0.0043-0.0062) | 1.8 case per 1000 patient per year | N/A^1^ |
| Ixekizumab | 6541(4 RCTs) | 14/5,191 (0.270%) | 0/1350 | MH RD 0.0027, (95% Cl 0.0001-0.0054) | 2.7 case per 1000 patient per year | N/A^1^ |
| Secukinumab | 1427(3 RCTs) | 4/843(0.474%) | 0/584 | MH RD 0.0046, (95% Cl -0.0027-0.0118) | 7.3 case per 1000 patient per year | N/A^1^ |
| Brodalumab | 3760(3 RCTs) | 1/2916 (0.034%) | 0/844 | MH RD 0.0003, (95% Cl -0.0027-0.0033) | 0.98 case per 1000 patient per year | N/A^1^ |
| Ustekinumab | 1664(4 RCTs) | 0/846 | 1/818(0.122%) | MH RD -0.0009，(95% Cl -0.0062-0.0043) | N/A^1^ | 1.1 case per 1000 patient per year |
| Total | 16,557 (21 RCTs) | 22/12,185(0.186%) | 1/4,372(0.022%) | MH RD 0.0017, (95% CI 0.0002-0.0032) | 2.4 case per 1000 patient per year | 0.3 case per 1000 patient per year |

1. Cannot estimate due to zero case reported in placebo group.
